# The serotonin-lir nervous system of the Bryozoa (Lophotrochozoa): a general pattern in the Gymnolaemata and implications for lophophore evolution of the phylum

**DOI:** 10.1186/s12862-015-0508-9

**Published:** 2015-10-14

**Authors:** Thomas F. Schwaha, Andreas Wanninger

**Affiliations:** Department of Integrative Zoology, University of Vienna, Althanstraße 14, 1090 Vienna, Austria

**Keywords:** Bryozoa, Serotonin, Gymnolaemata, Lophophore

## Abstract

**Background:**

Serotonin represents an evolutionary ancient neurotransmitter that is ubiquitously found among animals including the lophotrochozoan phylum Bryozoa, a group of colonial filter-feeders. Comparatively little is known on their nervous system, and data on their serotonin-lir nervous system currently are mostly limited to the basal phylactolaemates. Previous investigations indicated a common ground-pattern of the serotonin-lir nervous system in these animals, but in order to assess this on a larger scale, 21 gymnolaemate species from 21 genera were comparatively analysed herein.

**Methods:**

Twenty-one species from 21 gymnolaemate genera were analysed by immunocytochemical stainings and confocal laser scanning microscopy.

**Results:**

In all species the serotonin-lir signal is concentrated in the cerebral ganglion from where a nerve tract emanates laterally and traverses orally to engulf the foregut. Serotonin-lir perikarya are situated at the base of the tentacles that almost always correspond to the number of tentacles minus two. The oral side in almost all species shows three serotonin-lir perikarya followed by a ‘serotonergic gap’ that to our knowledge is not reflected in the morphology of the nervous system. Some species show additional serotonin-lir signal in tentacle nerves, visceral innervation and pore complexes. *Paludicella articulata* is exceptional as it shows signal in the latero-visceral nerves with serotonin-lir perikarya in the esophagus, parts of the tentacle sheath nerves as well as the frontal body wall around the parietal muscle bundles.

**Conclusions:**

In general, the serotonin-lir nervous system in the Bryozoa shows a consistent pattern among its different clades with few deviations. Preliminary data on phylactolaemates suggest the presence of a ‘serotonergic gap’ similar to gymnolaemates. Both show a subset of oral tentacles and the remaining tentacles in gymnolaemates which correspond to the lateral tentacles of phylactolaemates. The lophophoral concavity lacks serotonin-lir perikarya indicating that due to their larger sizes and increased tentacle number, the horse-shoe shaped arrangement could represent an apomorphy of phylactolaemates.

## Background

The transmission of neuronal signals among the Metazoa is commonly restricted to electrical or chemical signaling at the synapses between neurons [1]. Chemical neurotransmission involves neurotransmitters that activate a membrane-bound receptor on the postsynaptic side. Probably one of the oldest neurotransmitter that has been ubiquitously found among animals is serotonin or 5-HT (5-Hydroxytrypamin) [2]. In the last decades, the serotonin/serotonin-like compounds have been identified in various organisms and the distribution of the serotonergic nervous system in various organisms (often including entire developmental series) currently represents the largest dataset for any neuroactive compound (e.g. [3]).

The Bryozoa or Ectoprocta are a large group of sessile, colonial filter feeders with about 6000 recent species described. Despite their diversity, comparatively little is known of the nervous system of this phylum (e.g. [4, 5]). There are three distinct clades recognized among the Bryozoa: the Phylactolaemata, the Stenolaemata and the Gymnolaemata. The latter represents the largest and comprises about 5000 species. In all analysed species the nervous system consists of a cerebral ganglion at the lophophoral base wedged in between the mouth and anus. Circum-oral and circum– pharyngeal neurite bundles emanate from the ganglion and engulf the foregut. Additional nerves from the ganglion are the visceral nerves innervating part of the digestive tract and the tentacle sheath nerves that on their distalmost part reach into the body wall and consequently probably act in interzooidal communication [4, 5].

Most available data on the serotonergic-like (lir) nervous system of the Bryozoa are available on larvae [6–11]. A single analysis studied the metamorphic fate of the larval serotonin-lir nervous system as well as the newly-built juvenile condition of a gymnolaemate [12]. Adult specimens of the phylum have otherwise only been analysed in the Phylactolaemata [13, 14] and two gymnolaemate species [12, 15]. The Phylactolaemata has only few species and these are, with respect to zooid morphology, rather similar. Their serotonin-lir nervous system is present in the cerebral ganglion and at the lophophoral base its morphology is similar in all species studied so far (4 species of 2 genera, [13]; 1 additional genus & species in [14]; unpublished data on *Internectella bulgarica*; Schwaha pers. observations). The species-rich Gymnolaemata show an incredibly large variation concerning colony morphology as well as size and arrangement of individual zooids. The present data indicate that the serotonin-lir nervous system is similar among the Phylacto- and Gymnolaemata and that this possibly represents a conserved feature of the whole phylum. Given the few species studied (2 out of ~5000), the aim of this study is to analyse this part of nervous system in a broader spectrum and add several species of the Gymnolaemata in order to assess whether there is a common trend in the distribution of this part of the nervous system within the phylum.

## Methods

Twenty-one species from of 21 genera were collected from various localities (Sweden, Croatia, Thailand, Orkney Islands, Japan) by either dredging or from intertidal areas. None of the species are listed in any appendix of CITES (www.cites.org) and are not listed in the IUCN list of threatened species (http://www.iucnredlist.org). All procedures involving living animals were in strict accordance with national and international law (http://ec.europa.eu/food/animals/welfare/strategy/index_en.htm). The samples were fixed in 4 % paraformaldehyde in 0.1 M phosphate buffer (PB) (pH = 7.4) for 1 h at room temperature followed by 3 washing steps with the buffer. The samples were afterwards stored in 0.1 M PB containing 0.1 % NaN_3_. The calcified species were decalcified overnight with 50 mM EGTA solution. For permeabilization and blocking of unspecific binding sites, colony pieces were first cut into smaller pieces and then treated overnight with a 2–4 % Triton-X solution containing 6 % normal goat serum in 0.1 M PB (Block PBT). Then, the primary antibody, a polyclonal rabbit anti-serotonin (Zymed, San Francisco, CA, USA or Immunostar, Hudson, USA) was applied in Block PBT at a concentration of 1:500–1:1000. Unbound primary antibody was then eluted by several rinses in PBS followed by incubation with a goat anti-rabbit antibody coupled to AlexaFluor 488, 568 or 594 (Molecular Probes, Eugene, OR, USA) at a concentration of 1:300 in Block PBT overnight. Excessive secondary antibody was removed by several rinses in PBS. The samples were mounted in Fluoromount G (Southern Biotech, Birmingham, AL, USA) or Vectashield (Vector Laboratories, Burlingame, CA, USA). The samples were either analysed with a Leica SP2 or a SP5 II confocal laser scanning microscope (Leica Microsystems, Wetzlar, Germany). Data analysis was conducted with FIJI (www.fiji.sc) [16] or Amira (FEI, Hillsboro, Oregon, USA).

## Results and discussion

### General structure of the serotonin-lir nervous system and the distribution of other neuroactive compounds in the Gymnolaemata

**Fig. 1:**
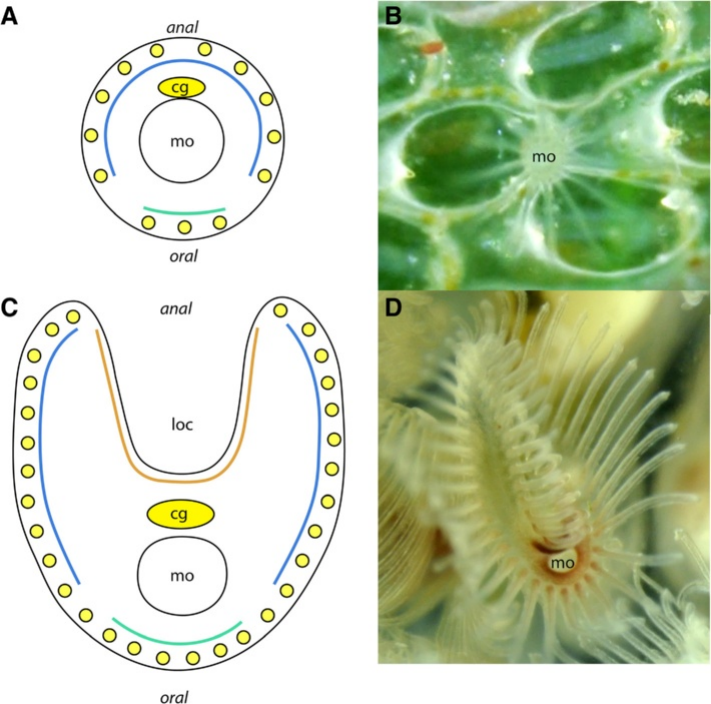
Schematic representation of the distribution of serotonin-lir perikarya in (**a & b**) the Gymnolaemata and (**c & d**) the Phylactolaemata. As shown in the current study the oral side of the Gymnolaemata always contains three serotonin-lir perikarya (turquoise) followed by a ‘serotonergic gap’ and the remaining lateral and anally situated tentacles (*blue*). Preliminary data on the Phylactolaemata hint to a similar arrangement of oral tentacles (turquoise), a kind of gap, followed by lateral tentacles rising up to the lophophore arms (*blue*). The inner lophophoral concavity shows no serotonin-lir perikarya. Consequently, it is possible that the inner row of tentacles (*orange*) represents an apomorphic feature of the Phylactolaemata. **b** is the cheilostome gymnolaemate *Electra posidonia*. **d** is the phylactolaemate *Pectinatella magnifica*. Abbreviations: cg – cerebral ganglion, loc – lophophoral concavity, mo – mouth opening

**Fig. 2:**
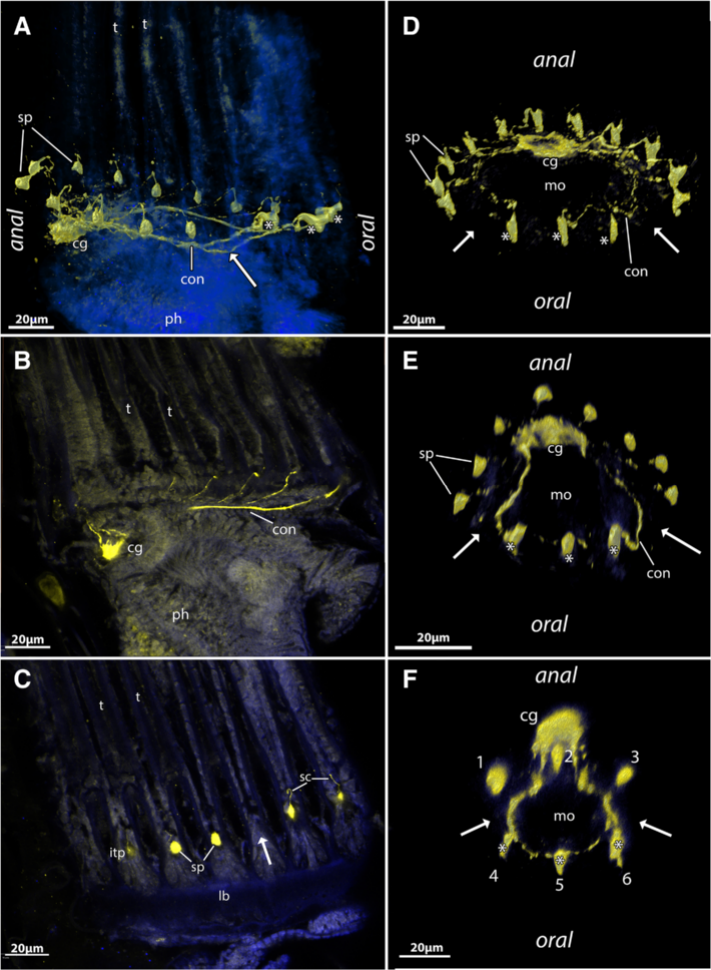
The general structure of the serotonin-lir nervous system in selected Gymnolaemata based on confocal laserscanning microscopy scans. **a-c** Serotonin-lir nervous system in the cheilostome *Membranipora membranacea*. **a** Volume rendering of the serotonin-lir nervous system (yellow) with nuclei (blue). View slightly oblique from the lateral side. The arrow points to the ‘serotonergic gap’ where the tentacle base does not possess a serotonin-lir perikaryon. **b** Optical section of the same specimen as in (**a**) showing the serotonin-like immunoreactivity in the cerebral ganglion. **c** Optical section of the same specimen as in (**a**) showing the series of serotonin-lir perikarya in the intertentacular pits including the ‘serotonergic gap’ (arrow). **d** Volume rendering of the serotonin-lir nervous system in the cheilostome *Cryptosula pallasiana*. The arrows point to the ‘serotonergic gap’. Asterisks mark the three oral serotonin-lir perikarya. **e** Volume rendering of the serotonin-lir nervous system in the cheilostome *Electra pilosa*. The arrows point to the ‘serotonergic gap’. Asterisks mark the three oral serotonin-lir perikarya. **f** Volume rendering of the serotonin-lir nervous system in the ctenostome *Amathia semiconvoluta*. This species has only 8 tentacles and consequently only 6 distinct serotonin-lir perikarya at the lophophoral base. The arrows point to the ‘serotonergic gap’. Asterisks mark the three oral serotonin-lir perikarya. Abbreviations: cg – cerebral ganglion, con – circum-oral nerve ring , itp – intertentacular pits, lb – lophophore base, mo – mouth opening, ph – pharynx, sc – serotonin-lir cilium(?), sp – serotonin-lir perikarya, t – tentacle

**Fig. 3:**
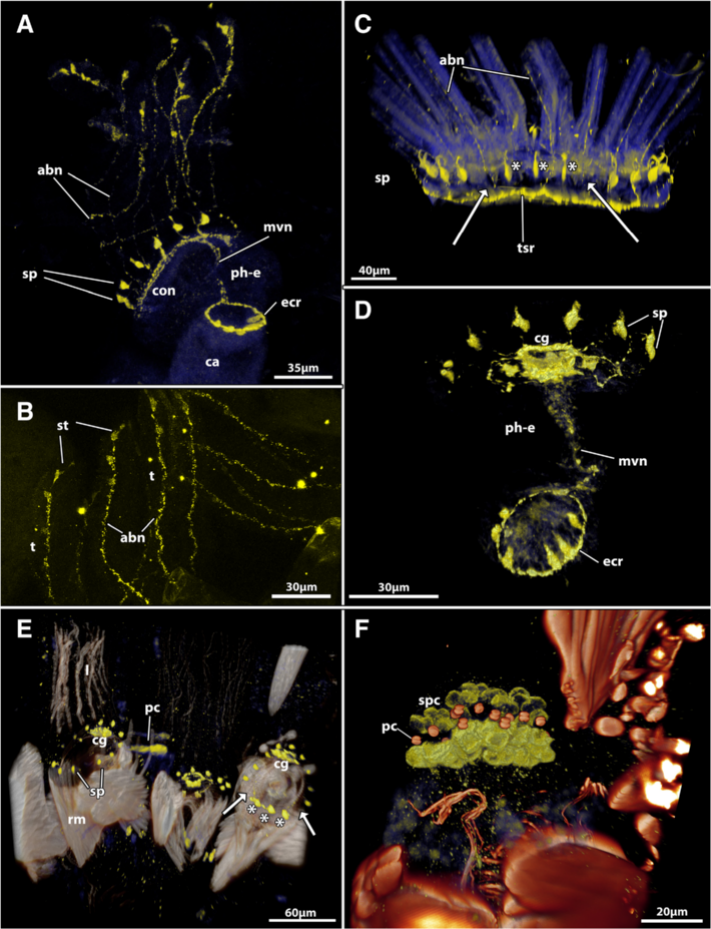
Deviations from the ‘common’ serotonin-lir nervous system found in the Gymnolaemata. **a & b** *Bicellariella ciliata* **a** Overview of the sertonin-lir nervous system with serotonin-lir abfrontal nerves and an additional serotonin-lir nerve ring at the esophagus-cardia border. Maximum intensity projection. **b** Detail of the tentacle tips in *B. ciliata* showing the sensory tips in the tentacles connected to the abfrontal nerve. Maximum intensity projection. **c** Volume rendering of the serotonin-lir nervous system of the ctenostome *Flustrellidra hispida*. In this species the abfrontal nerves show a faint staining and an additional nerve in the tentacle sheath is present. The arrows point to the ‘serotonergic gap’. Asterisks mark the three oral serotonin-lir perikarya. **d** The serotonin-lir nervous system of *Cribrilina annulata* shows the medio-visceral nerve and an additional serotonin-lir nerve ring at the esophagus-cardia border. From this ring 6 protruding, dent-like cells are distinguishable. **e & f** *Cellaria fistulosa*. **e** Volume rendering of the serotonin-lir nervous system of several zooids with musculature stained with phalloidin. The arrows point to the ‘serotonergic gap’. Asterisks mark the three oral serotonin-lir perikarya. **f** Detail of the serotonin-like immunoreactivity at the pore complexes showing distinct signal in the special cells. Abbreviations: abn – abfrontal nerve, ca – cardia, cg – cerebral ganglion, con – circum-oral nerve, ecr – nerve ring at the esophagus-cardia transition, mvn – mediovisceral nerve, pc – pore complex, ph-e – pharynx-esophagus, rm – retractor muscle, sp – serotonin-lir perikarya, spc – special cells, t – tentacle, tsr – tentacle sheath nerve ring

**Fig. 4:**
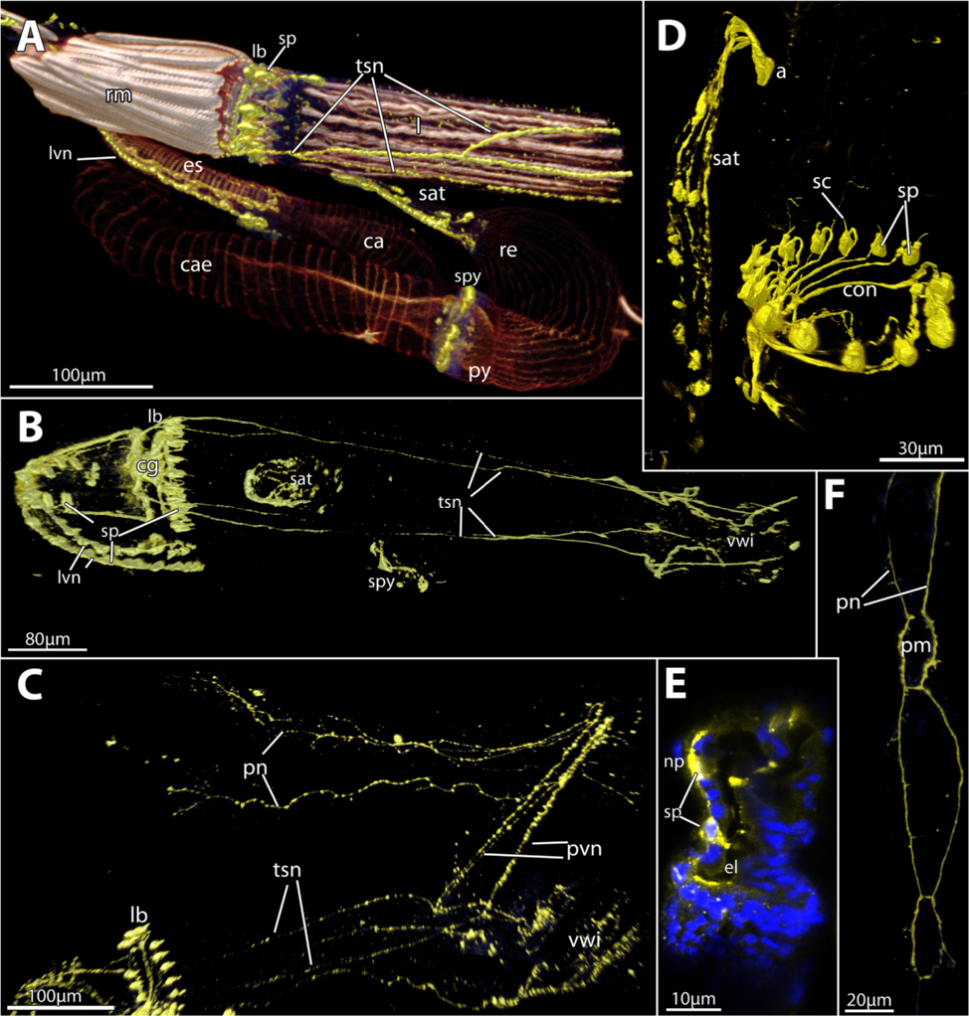
Volume rendering of the serotonin-lir nervous system of the ctenostome *Paludicella articulata*. **a** Overview of the polypide with the serotonin-lir nervous sytem (yellow) and the musculature (glow/red). **b** Detail of the serotonin-lir nervous system showing the concentration at the lophophoral base, the tentacle sheath nerves and the latero-visceral nerves innervating the foregut. **c** Parts of the serotonin-lir nervous system in a dissected zooid of *P. articulata* showing main parts of the serotonin-lir cystid innervation. **d** Close-up of the serotonin-lir concentration at the lophophoral base and the serotonin-lir anal tube. **e** Optical section through the esophagus showing the serotonin-lir perikarya within the lining of the esophagus. **f** Close-up of a parietal nerve innervating the parietal musculature. Abbreviations: a - anus ca – cardia, cae – caecum, cg – cerebral ganglion, con – circum-oral nerve ring, el – esophagus lumen, es – esophagus, lb – lophophore base, lvn – latero-visceral nerve, np – nucleus of the peritoneal lining, pm – parietal muscle, pn – parietal nerve, pvn – parieto-vaginal nerve, py - pylorus, re – rectum, rm – retractor muscles, sat – serotonin-lir anal tube, sc – serotonincilia, sp – serotonin-lir perikarya, spy – serotonin-lir pyloric ring, tsn – tentacle sheath nerve, vwi – vestibular wall innervation

In all 21 investigated species (Table 1; 8 Ctenostomata and 13 Cheilostomata) the serotonin-lir signal is highly concentrated in the cerebral ganglion which is situated next to the pharyngeal epithelium facing the side of the hindgut and anus (Figs. 1a, 2, 3d, e and 4b). On each lateral side of the ganglion a nerve tract emanates and traverses orally to engulf the foregut or pharynx. In all species distinct serotonin-lir perikarya are situated at the base of the tentacles within or at the distal border of the intertentacular pits (Figs. 1a, 2, 3a, c, d). The general organization of the serotonin-lir nervous system consequently corresponds to previous studies on the Phylactolaemata [10, 13, 14], the Ctenostomata [15] and a juvenile cheilostome [12]. The little data available on the FMRF-amidergic nervous system suggests a similar pattern in the phylactolaemates *Cristatella mucedo* and *Fredericella sultana* [14] as well as the cheilostome *Triphyllozoon mucronatum* [12]. In the phylactolaemates this specific nervous system extends beyond the cerebral ganglion and lophophoral base and is present in a tentacle nerve as well as the nerve plexus innervating the tentacle sheath [14]. The catecholamine-ergic nervous system was studied in *Cristatella mucedo* and appears to be distributed in the tentacles and the epistome [17]. Consequently, the distribution of neuroactive compounds is comparatively small in regard to the entire nervous system [4, 5]. The neuroactive compounds FMRF-amide and serotonin are the most widely studied among the metazoan phyla. Stainings for acetylated alpha-tubulin visualize most elements of the nervous system, but also cilia [18]. Particularly in retracted zooids the dense amount of cilia hinders or aggravates the analysis of the whole nervous system (Schwaha, pers. observation).

**Table 1:** List of gymnolaemate bryozoans investigated herein

	Species	No. of tentacles	Serot. perikarya	Abfrontal nerve	Peculiarities
Ctenostomata					
Alcyonidioidea	*Alcyonidium* sp.	15	13	**+/−**	
	*Flustrellidra hispida*	27	25	**+**	tentacle sheath ring
Arachnidioidea	*Nolella dilatata*	17	17	**–**	no ‘gap’
Paludicelloidea	*Paludicella articulata*	18	16	**–**	highly complex
Victorelloidea	*Victorella pavida*	8	6	**–**	
Walkerioidea	*Walkeria uva*	8	6	**–**	
Vesicularoidea	*Zoobotryon verticillatum*	8	6	**–**	
Vesicularoidea	*Amathia semiconvoluta*	8	6	**–**	
Cheilostomata					
Inovicellata	*Aetea anguina*	11	9	**+/−**	
Malacostega	*Membranipora membranacea*	17	15	**–**	
Malacostega	*Electra pilosa*	13	11	**–**	
Buguloidea	*Bugula purpurotincta*	15	13	**–**	
Buguloidea	*Bicellariella ciliata*	15	13	**+**	visc. nerve, cardiac nerve ring
	*Scrupocellaria scruposa*	12	10	**+**	
Cellarioidea	*Cellaria fistulosa*	14	12	**–**	pore-complex
Hippothoomorpha	*Chorizopora brongniartii*	12	10	**–**	
Cribrimorpha	*Cribrilina annulata*	13	11	**–**	visc. nerve, cardiac nerve ring
Lepraliomorpha	*Schizoporella* sp*.*	13	11	**–**	
Lepraliomorpha	*Cryptosula pallasiana*	16	14	**+**	
Lepraliomorpha	*Lanceopora* sp.	17	15	**–**	
Umbonulomorpha	*Reptadeonella violacea*	15	13	**–**

### Additional serotonin-lir components of the nervous system

In several species (see Table 1) an unpaired nerve extends from the basi-lophophoral perikarya into each tentacle (Fig. 3a-c). Frequently, the signal was not too intense or absent in some zooids of the respective species. Consequently this presence or absence was sometimes coded as +/− in the table. Since we observed only adult, functional zooids we exclude the possibility that the staining is related to developmental stage of the zooid. The nerve is on the outer side of the tentacle, i.e. represents the abfrontal nerve that is present in all Bryozoa (e.g. [5, 19, 20]). The exact number of tentacle nerves has been described from 4–6 (e.g. [15, 21]), but increasing evidence suggests four to be the ground-state at least for the Gymnolaemata [18]. Beside the abfrontal nerve, there are the two lateral-frontal and the median, frontal nerve on the inner side of the tentacle. The latter three are associated with ciliary bundles over the whole range of each tentacle that are required for creating feeding currents (e.g. [22]). The abfrontal nerve does not function in food uptake, but is equipped with (mostly ciliary) sensory structures (see [23]). In *B. ciliata* the distal-most tip even shows a concentration of serotonin-lir signal (Fig. 3b). A similarly shaped sensory cell at the tentacle tip was noted in the ctenostome *Flustrellidra hispida* [24], confirming the sensory nature of this nerve.

The cheilostomes *Bicellariella ciliata* and *Cribrilina annulata* show a serotonin-lir, unpaired visceral nerve that extends proximally on the pharynx until the border to the cardia. There, it forms a separate nervous ring (Fig. 3a, d). A ring nerve at the esophagus-cardia border has hitherto not been described in any species. From the latter, six serotonin-lir cells protrude medially and most likely constitute sensory epithelial cells of the digestive tract. Prominent visceral nerves seem to be common in all described Gymnolaemata (e.g. [4, 5, 18, 24], Schwaha unpubl. results). The stained nerve corresponds to the medial-visceral nerve of gymnolaemates. It appears that the visceral nerves commonly terminate at the esophagus-cardia junction, while the remaining digestive tract in general shows no concentrated neurite bundles, but single up to few individual nerve fibres [5, 25]. This seems to correlate with the amount of musculature associated with the various parts of the digestive tract, since the foregut is highly muscular with a common myoepithelial suction-pump of the pharynx, whereas the remaining digestive tract for the most parts contains a mere loose network of predominantly circular muscles in the Gymnolaemata [15].

The cheilostome *Cellaria fistulosa* shows additional staining for serotonin in the pore plate complex (Fig. 3e, f). Pore complex structures are a common feature of the Gymnolaemata with special cells responsible for interzooidal communication [26]. Particularly the Cheilostomata commonly possess so-called multiporous septulae, ie. pore complexes with multiple small communication pores contrary to the single-pored complex of most ctenostomes [5]. In *C. fistulosa* the cells that surround the small pores show an intense serotonin-lir signal in form of two cups on each side of the septula (Fig. 3f). The position and size corresponds to the so-called special cells of the pore-complexes [5, 26, 27]. The special cells are dumb-bell shaped and stretch through the thin pore complexes whereas their proximal and distal parts are swollen. In their differentiated form they show a certain polarity with the nuclei commonly being restricted to the proximal side [26, 27]. Also, in respect to the serotonergic-lir signal in *C. fistulosa*, the proximal area always shows a stronger signal (Fig. 3f). Possibly, this increased signal can be linked to a higher synthetic activity at the proximal side where the nuclei are located. Whether or not this concentration gradient has any significant effect on communication between zooids in the colony remains unknown. In the median plane of the pore complexes an enrichment of F-actin seems to be responsible for closure of the pores which corresponds to the cincture cells of other gymnolaemates [5, 27]. As described for other cheilostomes [27], there is always a single special cell associated with each pore (Fig. 3f, compare serotonin-lir and f-actin signal).

In the ctenostome *Flustrellidra hispida* a seronergic-lir ring nerve is present in the tentacle sheath (Fig. 3c). This ring nerve of the tentacle sheath has previously been described in this species [24], but has otherwise not been described in any other bryozoan [4]. Whether the nerve is present in other species but simply lacks serotonin-lir remains unknown.

### The complexity of *Paludicella articulata*

The freshwater ctenostome *Paludicella articulata* shows the ground-pattern of the serotonin-lir nervous system described above. However, the serotonin-lir immunoreactivity is much more complex than in all other species. There are two additional neurite bundles emanating from the cerebral ganglion (Fig. 4): 1) A pair of visceral nerves extends proximally on the digestive tract to the esophageal-cardiac border (Fig. 4a, b). In the traverse of the visceral nerves are serially repeated perikarya (Fig. 4e). About 13 perikarya were accounted in the epithelium of the foregut. So far, no serially repeated neuronal structures were encountered in any bryozoan.

In contrast to *B. ciliata* and *C. annulata*, the latero-visceral nerves [5, 18], and not the medio-visceral one, show positive signal. 2) One neurite bundle emanates from the cerebral ganglion and runs into the tentacle sheath. This bundle splits one or two times to form four serotonin-lir bundles within the tentacle sheath (Fig. 4a-c). On the one side, these nerves continue into the vestibular wall and on the other side traverse along the four parieto-vaginal bands towards the cystid wall (Fig. 4b, c). The immunoreactive signal continues from the parieto-vaginal bands only on the frontal side. Up to two serotonin-lir nerves are present on the frontal side and run over most of the length of the cystid wall. These surround the insertion part of each parietal muscle bundles (Fig. 4c, f) – gymnolaemate-specific muscles for polypide protrusion (see [28]). The ‘colonial’ nervous system or plexus (Hiller’s plexus) that innervates the cystid wall and forms the nervous connections between the zooids has been described in few gymnolaemates [29–34].

In addition to the nerves on the foregut there is a nerve-ring at the transition of the stomach to the rectum or hindgut. This area corresponds to the region referred to as pylorus which is commonly ciliated and causes rotation of the food particles in the stomach/caecum [35]. The ring is mainly composed of few serotonin-lir cell bodies within the epidermal layer (Fig. 4a, b). Likewise, the rectum shows several serotonin-lir perikarya and neurites embedded in its distal end that run in longitudinal direction (Fig. 4a, d). The latter two structures have to our knowledge not been described in any other bryozoan species before. Also, the link to the remaining nervous system remains elusive.

### The basi-lophophoral perikarya and the structure of the lophophore

**Fig. 5:**
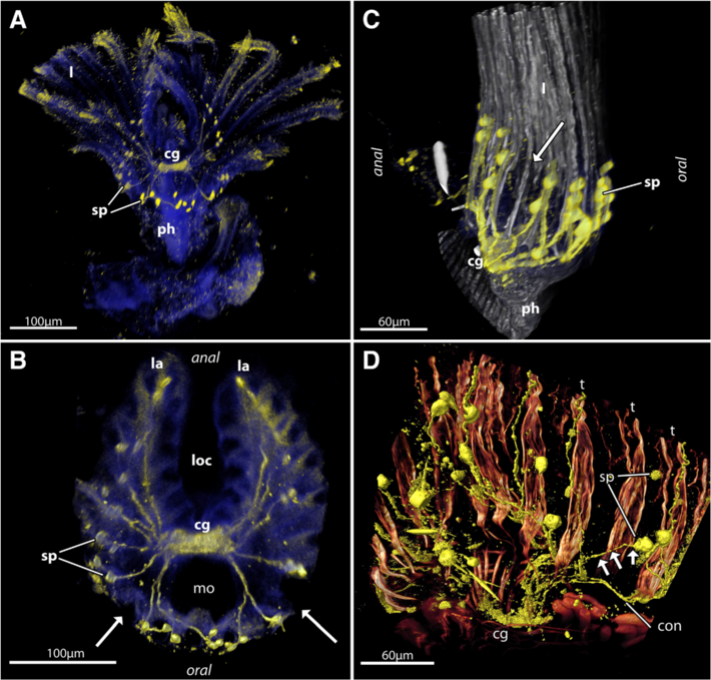
Serotonin-lir nervous system of different phylactolaemate bryozoans. **a & b** *Hyalinella punctata*. **a** Overview of serotonin-lir nervous system at the base of the lophophore. **b** Close-up of the serotonin-lir nervous system of the same specimen as in (**a**). The arrows point to a gap in the serotonin-lir signal similar to the gap observed in the Gymnolaemata. **c** Serotonin-lir nervous system of *Fredericella sultana* including phalloidin-staining to show general structure of the lophophore. The arrow points to a tentacle that lacks serotonin-lir perikarya at its base. **d** Detail of the serotonin-lir nervous system and musculature of the lophophoral base of *Stephanella hina*. The arrows point to a neurite bundle running to a serotonin-lir perikaryon at the tentacle base that derives from the neighbouring tentacle and not the circum-oral nerve ring. . ﻿cg - cerebral ganglion, con - circumoral nerve ring, l - lophophore, la - lophophore arms, loc - lophophoral concavity, mo - mouth opening, ph - pharynx, sp - serotonin-lir perikarya, t - tentacle

With the exception of *Nolella dilatata*, the number of serotonin-lir perikarya at the lophophoral base does not correspond to the amount of tentacles, but is always the number of tentacles minus two. On the oral side opposite to the anally situated ganglion there is a chain of three serotonin-lir perikarya followed by a gap with respect to the serotonergic immunoreactivity followed by the remaining tentacles towards the anal side (Fig. 1a, 2, 3c, e). This feature is easily overlooked in species with many tentacles and varying tentacle number (*Hislopia malayensis* [15], also present in this species), but becomes most evident in species with eight tentacles (Fig. 2f, Table 1). Currently, there is no functional explanation for this ‘serotonergic gap’. Neither the nervous system at the lophophoral base [4, 20] nor the intertentactular pits ([20], Schwaha pers. observations) where the perikarya are situated show any corresponding gap. It is possible that the gap is reflected in the ontogeny of the polypide. In all Bryozoa the polypide develops from a budding process. In the Gymnolaemata the lophophore forms as two lateral ridges that first fuse on the oral side and later on the anal one to form the circular adult form [20]. A similar process is found in the Phylactolaemata where two lateral bulges of the lophophore anlage protrude medially to form the orally situated tentacle row. It is conceivable that the gap in the Gymnolaemata is the border to the orally situated tentacle row. This notion implies that the Phylactolaemata also show this ‘serotonergic gap’ at the lophophoral base. However, in the species analysed so far this was not found [13, 14]. Species of the genus *Plumatella* sp. commonly possess numerous tentacles ranging from 25–60 [36]. In addition, the serotonergic nervous system is not restricted to single perikarya at the base of the tentacles, but commonly to 2–3 interconnected perikarya (personal observation from previous data of [13] as well as unpublished data). Thus, any possible gap is consequently easily overlooked. Owing to the less complex lophophore structure and less tentacles, *Fredericella sultana* commonly possesses single serotonin-lir perikarya at the lophophoral base [13]. Preliminary observations on this issue on the phylactolaemates *Fredericella sultana*, *Hyalinella punctata* and *Stephanella hina* indicate that gaps in the series of the serotonin-lir perikarya can be present as well. In the former two species there is a real ‘serotonergic gap’ sometimes present (Fig. 5a-c), whereas in *S. hina* the serotonin-lir perikaryon at the base of one tentacle is connected with the neighbouring neurite (Fig. 5d). As mentioned, these observations are preliminary and so far it is uncertain whether they occur in all specimens of the above-mentioned species. Nonetheless, when comparing the distribution of the serotonin-lir perikarya, two main subsets can be addressed: the oral perikarya and the lateral rows (Fig. 1). The lophophore is horse-shoe shaped in the Phylactolaemata and thus differs from the circular lophophore of all other Bryozoa – the Gymnolaemata and Stenolaemata. The inner lophophoral concavity lacks any serotonin-lir neurites or perikarya [13] and indicates that only the lateral tentacles showing serotonergic immunoreactivity are homologous to the lateral and anally situated tentacles of the Gymnolaemata (see Fig. 1). Following this line of thought, the tentacles on the inner lophophoral concavity would represent an apomorphic feature of the Phylactolaemata.

In the gymnolaemate ctenostome *Hislopia malayensis* young buds already possess the full tentacle number as in fully differentiated polypides [20]. In general, the tentacle number in the Gymnolaemata and the Stenolaemata rarely exceeds 18–20 tentacles. Although this needs to be confirmed in species with higher tentacle number, it is likely that all Gymnolaemata develop the full tentacle number during budding. Ontogenetically young zooids in the Phylactolaemata always possess comparatively few tentacles whereas fully grown zooid can have 70 or more tentacles [36]. Even in young buds, but also in juveniles, tentacle development takes place in the lophophoral concavity [15, 37]. Zooids of the Phylactolaemata are distinctly larger than any marine representatives (e.g. [5, 38]). Previously, phylactolaemaete bryozoans were regarded closely related to phoronids, in particular because of their similarly horse-shoe shaped lophophore (e.g. [39]). The most recent molecular phylogenies support lophophorate monophyly again with phoronids being sister-group to Bryozoa [40]. In regard to the arrangement of serotonin-lir perikarya and considering the phylactolaemate lophophore as apomorphic (see above), it seems more likely that the horse-shoe shape is a convergent development and perhaps correlates with the large size of the zooids in the Phylactolaemata. An alternative explanation would be that non-phylactolaemate Bryozoa (Steno- and Gymnolaemata) have lost the horse-shoe shaped lophophore.

## Conclusions

The current study is the first to analyse the serotonin-lir nervous system on a broader scale including 8 ctenostome and 13 cheilostome genera. It shows that the serotonin-lir nervous system in the Bryozoa seems to show a consistent pattern among its different clades. In comparison to the Phylactolaemata the contribution of the analysed Gymnolaemata yields new insight into the general lophophore structure and its possible evolution within the phylum. However, the Stenolaemata (Cyclostomata) remain to be studied on a broader scale in order to see whether they follow the pattern observed in the Phylactolaemata and Gymnolaemata.

Some species show additional serotonin-lir elements, but the significance of these in terms of functional or evolutionary interpretation remains unknown. The condition of *Paludicella articulata* is consequently unique among all bryozoan species studies so far, since the immunoreactivity against serotonin as well as FMRF-amide appears to be chiefly restricted to the cerebal ganglion and the circum-oral nerve ring [12–15]. Given its distribution mainly in sensory structures (lophoporal base sensory organs, abfrontal sensory nerve), it seems that serotonin for the most part is employed in sensory transductions in Bryozoa.
